# The Effect of SOD1 Mutation on Cellular Bioenergetic Profile and Viability in Response to Oxidative Stress and Influence of Mutation-Type

**DOI:** 10.1371/journal.pone.0068256

**Published:** 2013-06-28

**Authors:** Katie Richardson, Scott P. Allen, Heather Mortiboys, Andrew J. Grierson, Stephen B. Wharton, Paul G. Ince, Pamela J. Shaw, Paul R. Heath

**Affiliations:** Sheffield Institute for Translational Neuroscience (SITraN), University of Sheffield, Sheffield, United Kingdom; Baylor College of Medicine, Jiao Tong University School of Medicine, United States of America

## Abstract

Amyotrophic Lateral Sclerosis (ALS) is a fatal neurodegenerative disorder characterized by the progressive degeneration of motor neurons. Substantial evidence implicates oxidative stress and mitochondrial dysfunction as early events in disease progression. Our aim was to ascertain whether mutation of the SOD1 protein increases metabolic functional susceptibility to oxidative stress. Here we used a motor neuron-like cell line (NSC34) stably transfected with various human mutant *SOD1* transgenes (G93A, G37R, H48Q) to investigate the impact of oxidative stress on cell viability and metabolic function within intact cells. NSC34 cells expressing mutant SOD1 showed a dose dependent reduction in cell viability when exposed to oxidative stress induced by hydrogen peroxide, with variation between mutations. The G93A transfectants showed greater cell death and LDH release compared to cells transfected with the other *SOD1* mutations, and H48Q showed an accelerated decline at later time points. Differences in mitochondrial bioenergetics, including mitochondrial respiration, coupling efficiency and proton leak, were identified between the mutations, consistent with the differences observed in viability. NSC34 cells expressing G93A SOD1 displayed reduced coupled respiration and mitochondrial membrane potential compared to controls. Furthermore, the G93A mutation had significantly increased metabolic susceptibility to oxidative stress, with hydrogen peroxide increasing ROS production, reducing both cellular oxygen consumption and glycolytic flux in the cell. This study highlights bioenergetic defects within a cellular model of ALS and suggests that oxidative stress is not only detrimental to oxygen consumption but also glycolytic flux, which could lead to an energy deficit in the cell.

## Introduction

Amyotrophic Lateral Sclerosis (ALS) is a fatal neurodegenerative disorder, characterized by the progressive degeneration of upper and lower motor neurons [1], [2]. The majority of ALS cases are sporadic but around 10% are familial in origin [3]. Cu/Zn Superoxide Dismutase 1 (*SOD1*) was the first gene identified as mutated in ALS [4], and accounts for approximately 2% of all ALS cases [5], [6]. SOD1 is a ubiquitously expressed metalloenzyme that catalyses the dismutation of superoxide radicals into hydrogen peroxide and molecular oxygen. Mutant *SOD1* is toxic by a mechanism that is independent of its dismutase activity [7]. The identification and characterization of mutations in *SOD1* has led to the development of *in vitro* and *in vivo* models from which have emerged much of our current understanding of the pathophysiological mechanisms involved in ALS [8].

Mitochondria form highly dynamic networks and are the primary site of ATP production through oxidative phosphorylation [9], [10]. These metabolic reactions are major sources of reactive oxygen species (ROS), which can inflict damage to macromolecules. A characteristic manifestation of neurodegenerative disease is mitochondrial dysfunction and studies from both ALS patients and transgenic animal models have demonstrated changes in mitochondrial function including a reduction in the activity of respiratory chain complexes and decline of mitochondrial bioenergetic capacity [11], [12], [13]. A functional compromise to energy production results in a loss of mitochondrial membrane potential, in addition to impaired electron chain transport activity and a reduction in ATP production, with an accompanying increase in ROS production [13], [14], [15]. *In vitro* studies have shown that the transfection of human mutant *SOD1* variants can cause a shift in redox potential, an increase in mitochondrial superoxide dismutase levels, increased toxicity, and a reduction in respiratory chain complex activity [16], [17], [18]. *In vivo* murine studies have also demonstrated the vulnerability of mitochondria together with disturbances to calcium homeostasis in the presence of human G93A mutant *SOD1* [19], [20]. Although mitochondrial impairment and increased oxidative stress have been extensively documented in models of ALS, the causal relationship between impaired bioenergetics, physiological malfunction, and oxidative damage needs to be further established in relation to the underlying pathogenesis of ALS.

In this study we utilised the murine neuroblastoma motor neuron (NSC34) hybrid cell line [21], stably transfected with human mutant *SOD1* transgenes. *SOD1* mutations in these cell lines have previously been shown to increase oxidative stress and mitochondrial dysfunction, with key genes down regulated in their metabolic pathways [14], [22]. We expressed wild-type human *SOD1* (WTSOD1), G93A mutant human *SOD1*, H48Q mutant human *SOD1*, G37R mutant human *SOD1*, and a pIRES vector control in the NSC34 cell line and determined the effect on the susceptibility to oxidative stress in terms of cell viability, mitochondrial and metabolic function. We show that expression of human G93A and H48Q *SOD1* mutations renders neuronal cells more susceptible to oxidative stress in terms of viability and significant differences in mitochondrial bioenergetics are identified in the G93A mutant *SOD1* cells compared with the controls and other mutations investigated, both under basal and stress conditions.

## Methods

### Cell Culture

NSC34 mouse motor neuron cells [21] (a gift from Dr Neil Cashman, University of British Columbia, Canada) were stably transfected with pIRESneo (Clontech, Saint-Germain, France) using Lipofectamine 2000 (Invitrogen). Cells were transfected with empty vector (pIRES cells) or pIRESneo containing wild-type human *SOD1* (WTSOD1 cells), human *SOD1* mutant G93A, human *SOD1* mutant G37R, or human *SOD1* mutant H48Q. Geneticin (G418, Invitogen) at 250 µg/ml was added to cells 24 hours later to select for transfected cells. The expression levels for the human *SOD1* transgenes were determined by real time quantitative PCR (RT-qPCR) as described elsewhere [22]. The level of human *SOD1* in each of the cell lines was determined by measuring the difference in the deltaCt between the human *SOD1* and mouse *Sod1* transcripts [23]. Western blotting was used to determine the protein levels of human and mouse SOD1 in the NSC34 cells using a sheep anti-*SOD1* (Cu/Zn) polyclonal antibody (1∶1000, Calbiochem, Nottingham, UK).

### Cell Viability Assay

NSC34 pIRES vector control, WTSOD1, G37R, H48Q and G93A cells were plated into 6 cm diameter petri-dishes and cultured under normal growth conditions in DMEM 4.5 mg/ml glucose (Sigma) supplemented with 10% Biosera Fetal Calf Serum, 2 mM glutamine (Lonza) and G418 (250 µg/ml) at 37°C/5% CO_2_, until they reached 70–80% confluency. Cells were treated with hydrogen peroxide (H_2_O_2_) at concentrations ranging from 50 µM to1 mM for two, four, six, and ten hours. The assay was performed under normal growth conditions and the effect of oxidative insult on cell viability was measured using 0.4% trypan blue dye exclusion (Sigma) and a Coulter Cell Counter (Invitrogen).

### Lactate Dehydrogenase Assay

NSC34 pIRES vector control, WTSOD1, G37R, H48Q and G93A cells were plated into 96 well plates and incubated for 24 hours under normal growth conditions in DMEM 4.5 mg/ml glucose (Sigma) supplemented with 10% Biosera Fetal Calf Serum, 2 mM glutamine and G418 (250 µg/ml) at 37°C/5% CO_2_. Cells were treated with 250 µM, 500 µM and 1 mM H_2_O_2_ for two, four, six, and ten hours. The assay was performed under normal growth conditions and the effect of oxidative insult on lactate dehydrogenase (LDH) was measured. The culture medium was collected and LDH release was measured using a CytoTox 96® Non-Radioactive Cytotoxicity Assay (Promega, G1780). Briefly, post stress, 50 µl of culture media was removed and added to 50 µl of assay substrate mix, incubated at room temperature in the dark for 15 minutes then the absorbance was recorded at 490 nM on a Fluorostar Omega plate reader (BMG Labtech). Total LDH was assessed by freeze thawing the assay plate and repeating the procedure described above. Percentage LDH release was calculated using the following formula, % LDH release = (H_2_O_2_ induced LDH release/Total LDH)×100.

### Seahorse Metabolic Assay

NSC34 pIRES vector control, WTSOD1, G37R, H48Q and G93A cells were plated in a 24 well Seahorse cell culture plate (Seahorse Bioscience, 100777-004) in 250 µl DMEM 4.5 mg/ml glucose supplemented with 10% Biosera fetal calf serum, 2 mM glutamine and 250 µg/ml G418. The cells were incubated at 37°C/5% CO_2_ overnight. The following day the media was removed and replaced with XF assay media pH 7.4 (Seahorse Bioscience 101022-100) supplemented with 2 mM glutamine and 4.5 mg/ml glucose. Cells were incubated at 37°C for 1 hour. Meanwhile, a 24 well microplate was loaded with 5.0 µg/ml oligomycin (Sigma) final concentration 0.5 µg/ml, 2.50 µM carbonyl cyanide-p-trifluoromethoxyphenylhydrazone (FCCP) (Sigma) 0.25 µM final concentration, and 5.0 µM Rotenone (Sigma) final concentration 0.5 µM. All were made up in XF assay media as described above. The microplate was calibrated in a Seahorse XF24 analyzer prior to addition of the cell culture plate. Three basal measurements were recorded (three minutes each) prior to addition of oligomycin, FCCP and finally rotenone. The effect of the drugs on oxygen consumption and ECAR were measured three times (3 minutes each). Cell number was normalized by addition of 4.0 µM calcein (Invitrogen), which was incubated with the cells for 30 minutes and fluorescence measured on a Fluorostar Omega plate reader (BMG Labtech) at Ex485 nm/Em530 nm. For the stress assays, the cells were incubated with 50, 100 and 200 µM H_2_O_2_ for one hour at 37°C/5% CO_2_ prior to preparation of the cells for analysis on the XF 24 bioanalyser as described above.

### Mitochondrial Membrane Potential Assay

NSC34 cells were plated at 15,000 cells per well in a gelatin coated (0.5 mg/ml gelatin overnight at 4°C) 96 well plate in 200 µl DMEM 4.5 mg/ml glucose supplemented with 10% Biosera fetal calf serum, 2 mM glutamine and 250 µg/ml G418, then incubated at 37°C/5% CO_2_. 24 hours later, the media was removed and replaced with glucose free DMEM media (Lonza) +10% FCS +0.9 mg/ml galactose. After a further 23 hours, 10 µM FCCP in 40% ethanol was added to half the wells to dissipate the mitochondrial membrane potential. Cells were incubated at 37°C/5% CO_2_ for 1 hour, the media was then removed and the cells were incubated with 150 nM tetramethylrhodamine methyl ester (TMRM, Sigma) for 15 minutes at 37°C/5% CO_2_. Cells were then washed three times with 150 µl 1×PBS. The plate was read on a BMG Fluorostar plate reader at Ex544 nm/Em590 nm for TMRM fluorescence. Cell death was simultaneously measured by adding 0.3 µM ethidium homodimer-1(EthD1, Molecular Probes) to the culture medium and measuring fluorescence at Ex530 nm/Em645 nm. TMRM fluorescence was normalized to cell number, determined by measuring EthD1 fluorescence (0.3 µM) after cells had been freeze–thawed. Mitochondrial membrane potential was calculated by subtracting the TMRM fluorescence in the presence of FCCP (plasma membrane potential contribution) from the fluorescence in the absence of FCCP.

### H_2_O_2_ Oxidative Stress Assay

NSC34 cells were grown in gelatin coated 96-well tissue-culture plates in phenol red-free DMEM (Lonza) until 70% confluent. 100 µM H_2_O_2_ was added to the cells for 60 minutes at 37°C. The media was subsequently removed and the cells were washed twice with 1×PBS. Cytosolic reactive oxygen species levels were measured using dichlorofluorescein (DCF) fluorescence. Carboxy-H2DCFDA (6-carboxy-2′,7′-dichlorodihydrofluorescein diacetate, di(acetoxymethyl ester);MolecularProbes, Paisley, UK) was added to the NSC34 cells at 10 µM, and the fluorescence of oxidized DCF was read at Ex485 nm/Em530 nm after 90 minutes using a BMG Fluorostar plate reader (BMG Labtech). Cell number was measured by adding EthD1 to the culture medium after the cells had been freeze–thawed. Fluorescence was measured at Ex530 nm/Em645 nm. Raw DCF data results were then normalized to cell number.

### Statistical analyses

For the cell viability assays, five replicates for each transfectant at each time point/concentration were performed and normalized to the viability of the untreated pIRES cells. The means/standard deviations were calculated and a two-way ANOVA by mutation at each concentration was performed using GraphPad Prism version 5.0d for Mac (GraphPad Software, La Jolla California USA). For each control and mutation, the metabolic assay was carried out four times under both basal conditions, with three technical replicates generated per experiment. All data were normalised to cell number prior to analysis. A one-way ANOVA with Bonferroni post-hoc test was performed using GraphPad Prism version 5.0d for Mac (GraphPad Software, La Jolla California USA) to investigate changes under basal conditions and stress conditions for each cell type individually. For each control and mutation the metabolic assay following oxidative stress was carried out six times, with three technical replicates generated per experiment. A two-way ANOVA by H_2_O_2_ dose was performed to investigate the effect of the stress on OCR/ECAR across the mutations. For the mitochondrial membrane potential analysis, the assay was performed seven times with a minimum of three replicates per experiment for each control and mutant. TMRM fluorescence in the presence of FCCP was subtracted from TMRM fluorescence in the absence of FCCP. All data were normalized to cell number prior to mitochondrial membrane potential calculation. A one-way ANOVA with Bonferroni post hoc analysis test was performed using Graph Pad Prism version 5.0d to investigate the effect of SOD1 mutation on the NSC34 cell mitochondrial membrane potential.

## Results

### Susceptibility of NSC34 cells expressing mutant SOD1 G93A to oxidative stress

**Figure 1:**
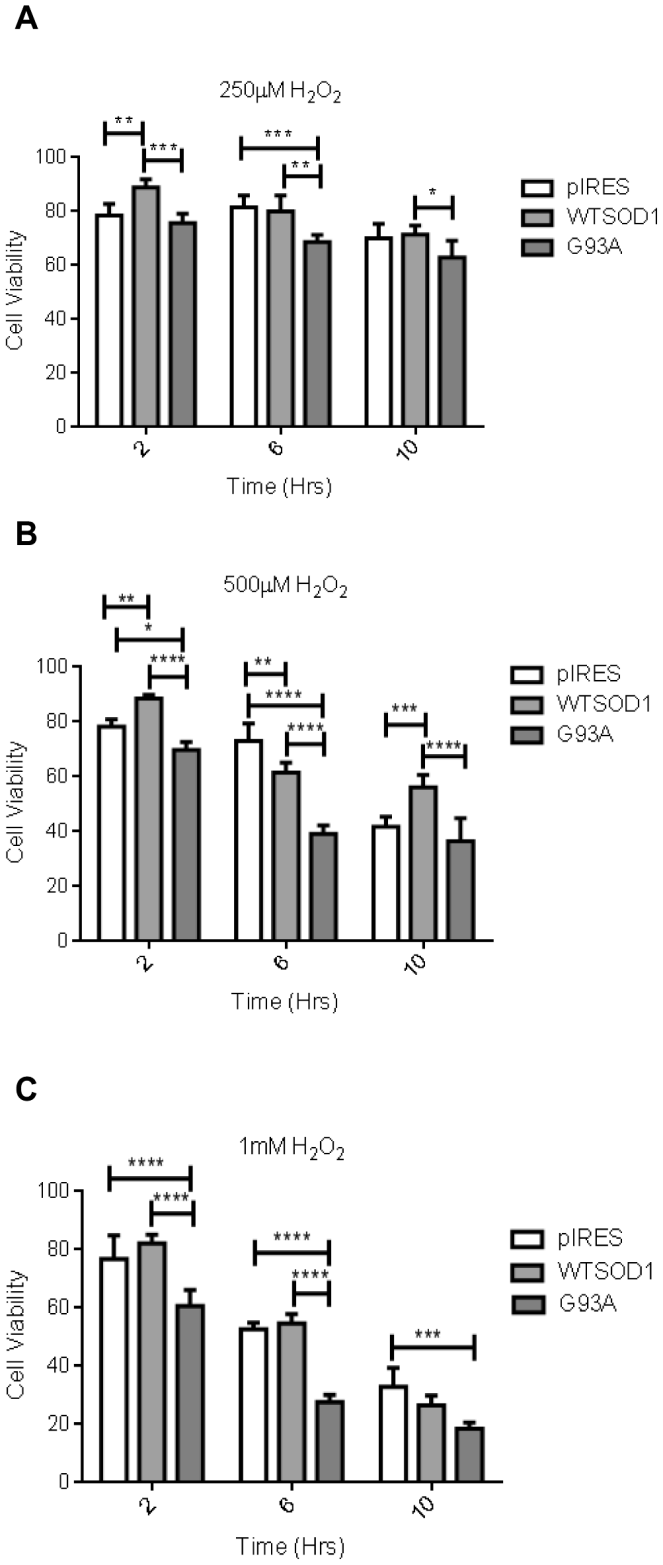
The effect of G93A SOD1 mutation on NSC34 cell viability. The G93A mutation was more susceptible to oxidative stress in terms of cell viability at A. 250 µM B. 500 µM and C. 1 mM. Cells were incubated for 2, 6 and 10 hours with H_2_O_2_ prior to cell counting. Data presented as mean with SD n = 5. Statistical analyses by two-way ANOVA with Bonferroni post-test, **** = P≤0.0001, *** = P≤0.001 ** = P≤0.01* = P≤0.05.

**Figure 2:**
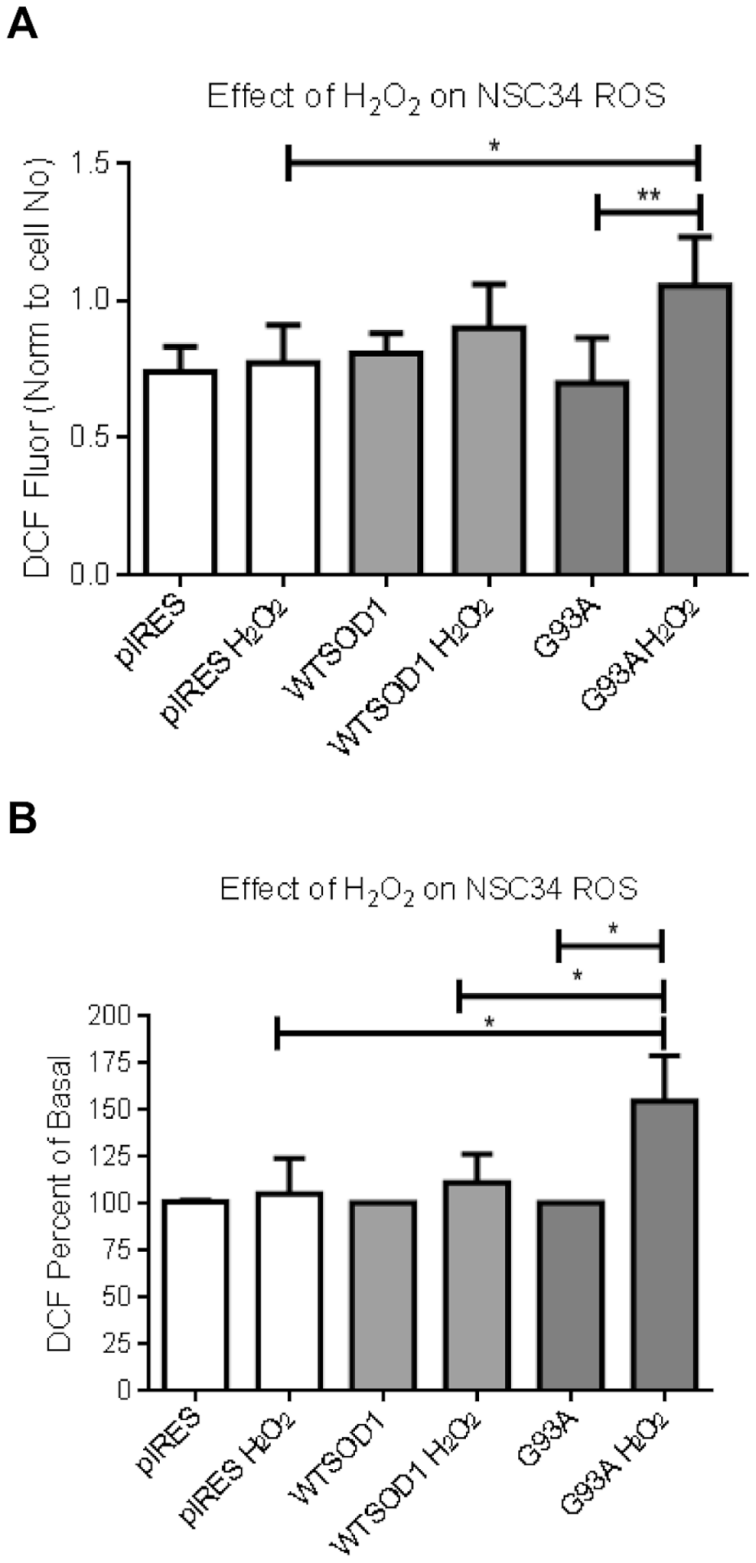
The effect of H_2_O_2_ on ROS levels in NSC34 cells. A. Raw DCF fluorescence. B. Data normalized to basal levels. Cells were treated with 100 µM H_2_O_2_ for one hour at 37°C. Cells were washed prior to addition of DCF for 90 minutes at 37°C. Data presented as mean with SD n = 5. Statistical analysis by A. One way ANOVA with Bonferroni post test. B. Kruskal Wallis test with Dunns post test, ** = P≤0.01, * = P≤0.05.

An *in vitro* model of SOD1-mediated ALS was previously established in our lab by generating single cell clones of NSC34 motor neuron cells stably expressing equivalent amounts of either normal human SOD1 or the G93A mutant SOD1 [24]. The G93A SOD1 mutant has WT dismutase activity but has increased free radical-generating function [25]. We have previously shown a significant increase in cytosolic oxidative stress in the NSC34 cells expressing human G93A SOD1 compared to cells expressing the pIRES vector or human WTSOD1 [24]. To ascertain whether mutation of the SOD1 protein led to metabolic susceptibility to oxidative stress, the effect of H_2_O_2_ on cell survival was investigated. Oxidative stress was induced for two, six and ten hours at 37°C and cell survival was assayed by trypan blue exclusion. Increasing H_2_O_2_ led to increased cell death across all cell lines investigated (Figure 1 A–C), with the G93A mutant SOD1 cells displaying the greatest susceptibility to H_2_O_2_ compared to control cells at all H_2_O_2_ concentrations (Figure 1 A–C). Further analysis of the G93A mutant SOD1 cells showed a significant increase in ROS levels above basal in the presence of H_2_O_2_ in comparison to the controls (Figure 2A). Furthermore when normalised to basal levels the percentage increase was significantly higher in the G93A SOD1 cells than controls (Figure 2B, p≤0.05).

### Characterization of mitochondrial function in NSC34 cells expressing mutant SOD1 G93A

**Figure 3:**
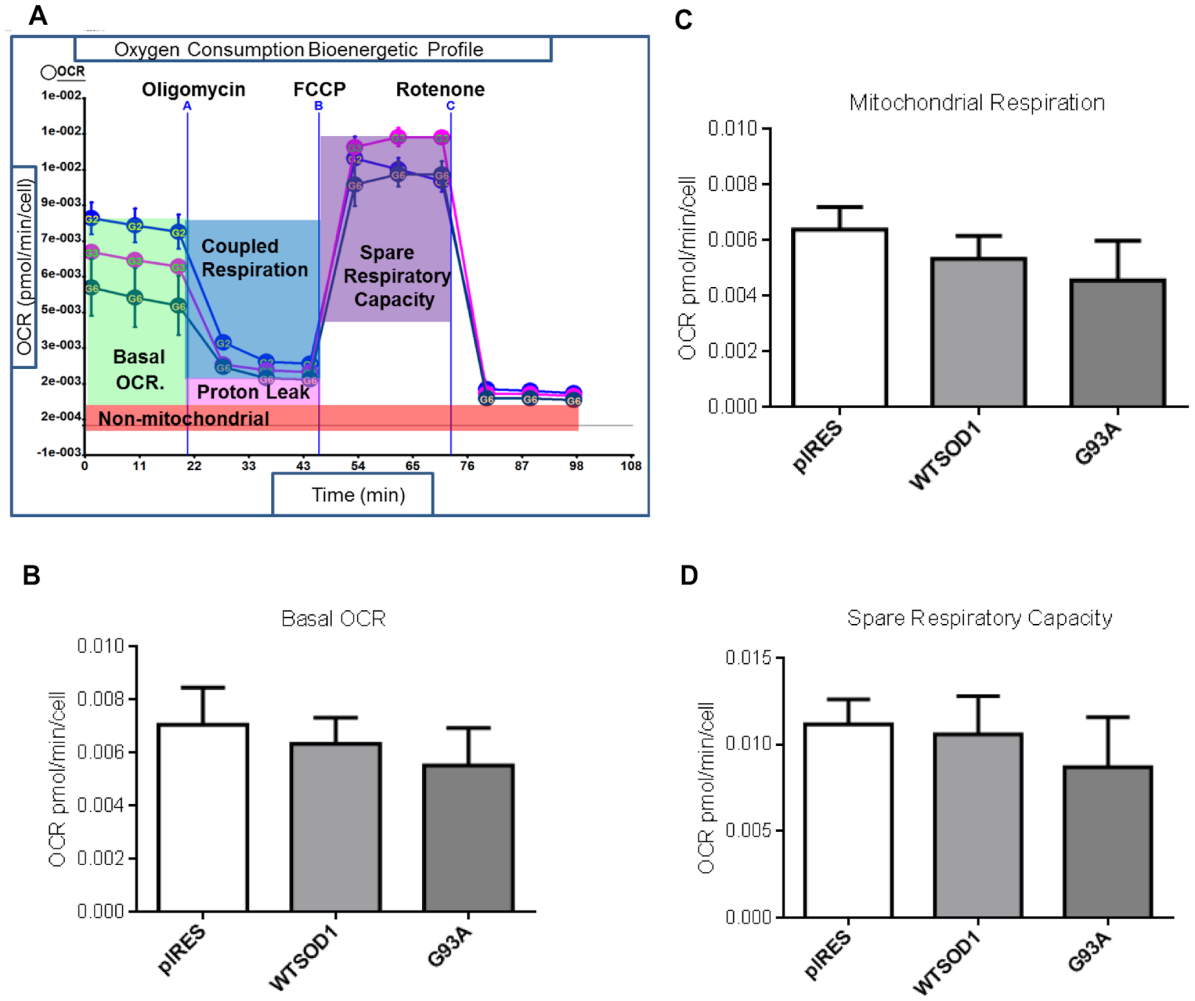
The effect of G93A SOD1 mutation on oxygen consumption. A. Representative OCR bioenergetic profile of NSC34 cells. Blue-pIRES, pink-WTSOD1, dark blue-G93A SOD1. B. Basal cellular oxygen consumption. C. Mitochondrial oxygen consumption. Mitochondrial respiration was calculated by subtracting OCR in the presence of rotenone from basal OCR. D. Spare respiratory capacity was calculated by subtracting maximal respiration from mitochondrial respiration.

With the aim of creating a bioenergetic profile of the neuronal cell model, the NSC34 cells were analysed on an XF-24 Extracellular Flux Analyzer (Seahorse Biosciences), which simultaneously monitors the two major energy-yielding pathways in cells, aerobic respiration and glycolysis, by measuring the oxygen consumption rate (OCR) and extracellular acidification rate (ECAR). Cellular respiration was assessed under basal conditions (basal respiration), with the ATP synthase inhibitor oligomycin (coupled respiration), the mitochondrial membrane uncoupler FCCP (to measure spare respiratory capacity) and the mitochondrial complex I inhibitor rotenone to assess mitochondrial specific respiration (Figure 3A). The sequential addition of these compounds shifts the bioenergetic profile of cells, allowing differences in mitochondrial function to be compared between cell lines.

Basal oxygen consumption rate (bOCR) is an indicator of both mitochondrial and non-mitochondrial respiration and is controlled strongly by ATP turnover and partly by substrate oxidation and proton leak [26], [27]. No significant difference in bOCR was observed between the G93A mutant SOD1 cells and controls (Figure 3B). The fraction of cellular oxygen consumption linked to the mitochondria (mOCR) was measured by addition of rotenone. No significant difference was observed between G93A SOD1 and controls (Figure 3C). The application of FCCP dissipates the proton gradient across the mitochondrial inner membrane and allows investigation of maximal mitochondrial respiration. When FCCP was added to induce maximal respiration, the calculated spare respiratory capacity of the G93A cells was reduced compared to controls, however the reduction was again non-significant (p>0.05) (Figure 3D).

**Figure 4:**
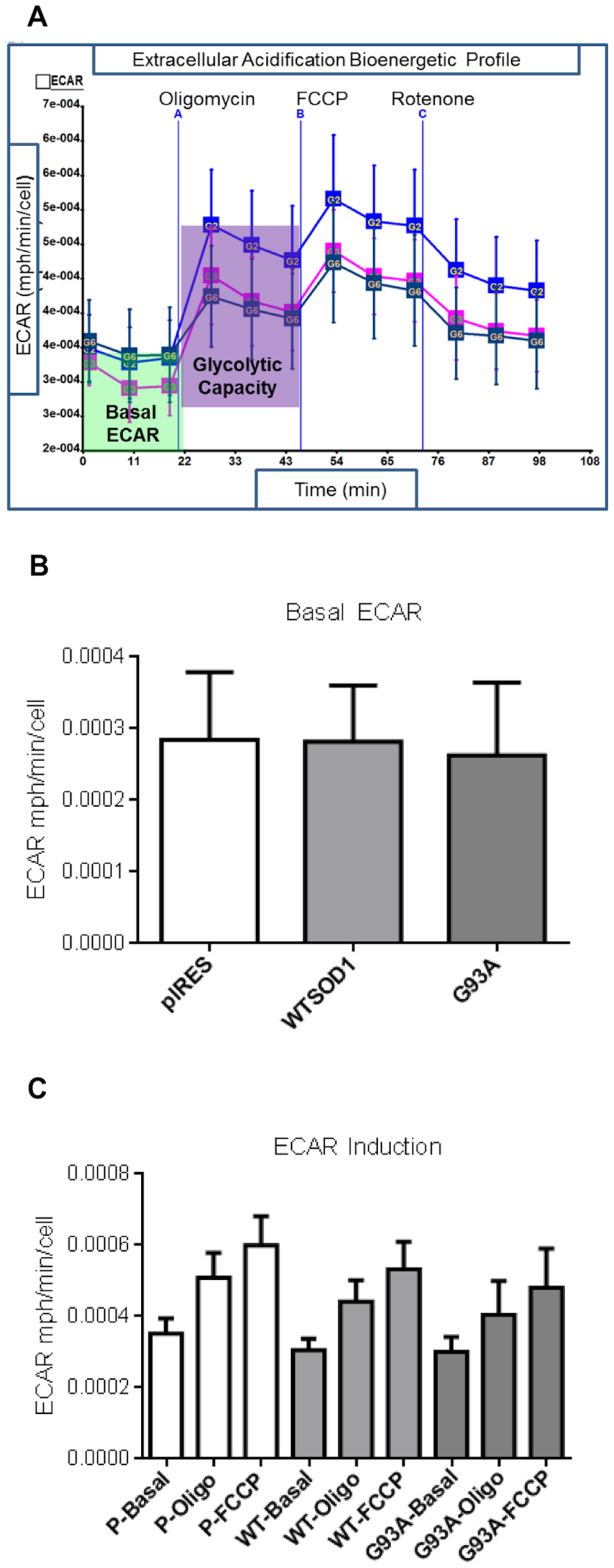
The effect of G93A SOD1 mutation on glycolytic flux. A. Representative ECAR bioenergetic profile of NSC34 cells. Blue-pIRES, pink-WTSOD1, dark blue-G93A SOD1. B. Basal ECAR. C. ECAR induction with addition of oligomycin (Oligo) and FCCP. P = pIRES. Data presented as mean with SD n = 5.

Together with measurements of aerobic respiration, the XF24 Seahorse bioanalyser also enables investigation of the extracellular acidification rate (ECAR), a direct measure of lactate produced by glycolytic flux. Moreover, the response of the glycolytic flux to mitochondrial inhibition can be measured (Figure 4A). When ATP synthase is inhibited, the cell responds by up-regulating glycolysis to recover the energy deficit; this increase above basal levels is termed the glycolytic capacity. A concomitant rise in ECAR is also observed when injecting FCCP, which is likely to be due to the contribution of bicarbonated CO_2_ to ECAR, produced by upregulation of the TCA cycle. In terms of basal ECAR, no difference was observed between G93A mutant SOD1 and control cells (Figure 4B). When analysing induction of the glycolytic flux, although glycolytic capacity was reduced by approximately 20% in the G93A cells it did not reach significance (p>0.05) (Figure 4C).

**Figure 5:**
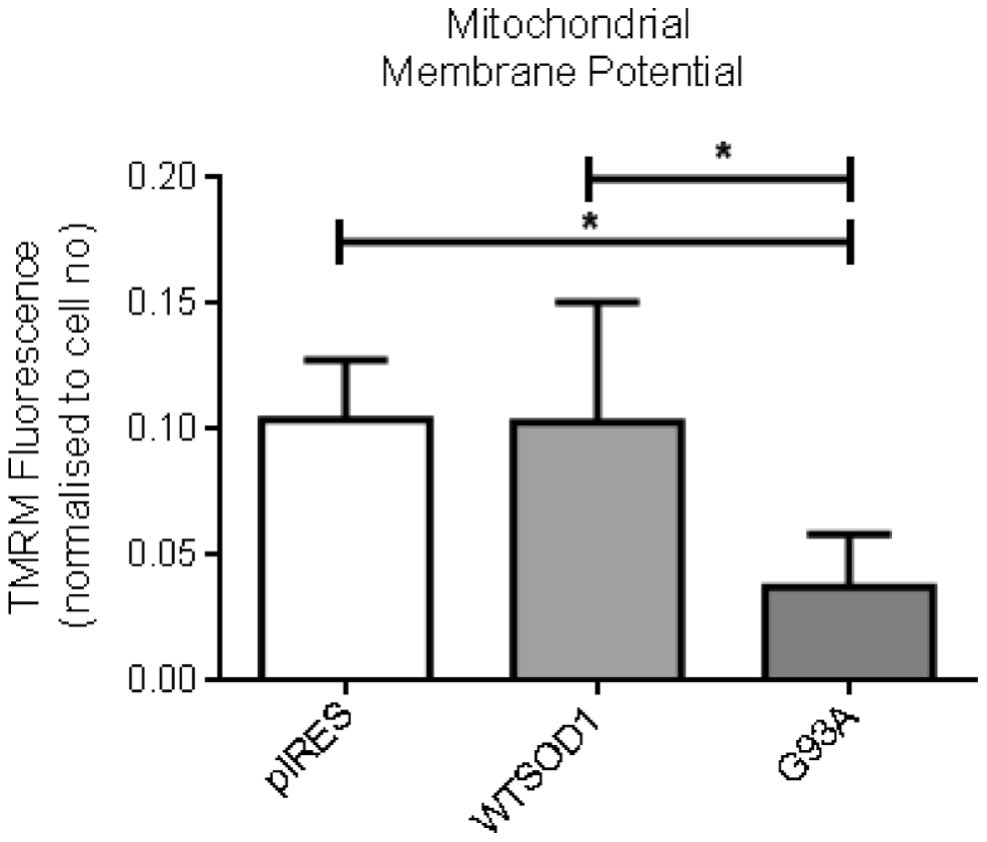
The effect of G93A SOD1 mutation on mitochondrial membrane potential. Membrane potential was measured using 150 nM TMRM for 15 minutes at 37°C −/+10 µM FCCP. Data presented as mean with SD n = 7. Statistical analyses by one-way ANOVA with Bonferroni post-test, * = P<0.05.

To further investigate mitchondrial dysfunction in the G93A cells, the mitochondrial membrane potential was measured using the fluoresecent dye TMRM. The G93A mutant SOD1 cells had lower mitochondrial membrane potential compared to the pIRES vector and WTSOD1 control cell lines (Figure 5, p≤0.05).

### Effect of oxidative stress on the cell viability of NSC34 cells expressing different mutant SOD1 transgenes

We now proceeded to investigate whether the susceptibility to oxidative stress varied between different *SOD1* mutations. We therefore carried out repeat experiments using the controls and G93A mutant SOD1 cells, alongside G37R and H48Q human mutant SOD1 transfected NSC34 cells. Previously published data from our laboratory showed equivalent human SOD1 protein expression levels in all transfected cell lines [24]. In addition, no significant differences at the level of transfection for the WTSOD1 and the mutant *SOD1* transgenes were detected, as determined by RT-qPCR (Figure S1), confirming equivalent transfection through transcription.

**Figure 6:**
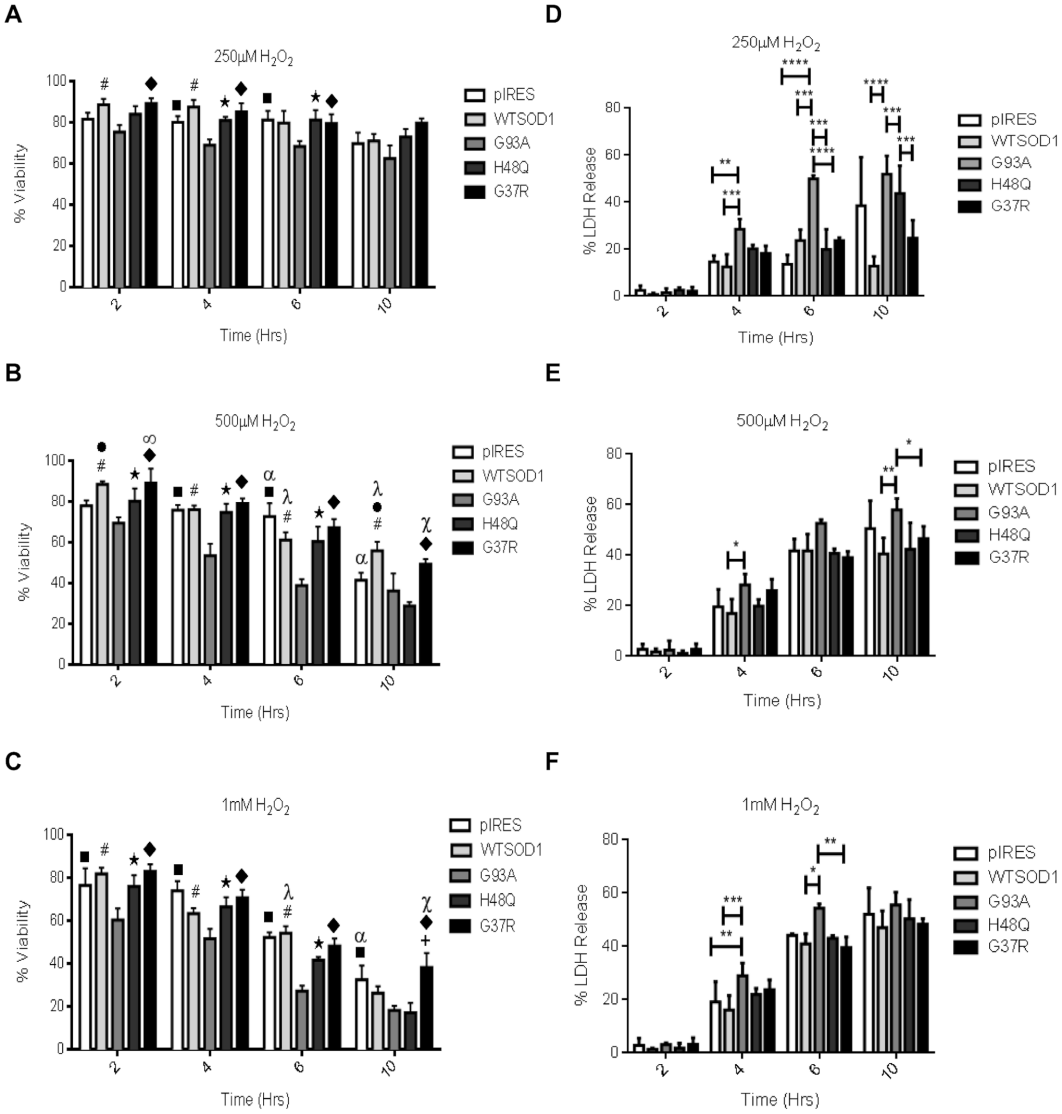
The effect of SOD1 mutation on NSC34 cell viability. A-C. Trypan blue exclusion assays. The G93A mutation was the most susceptible to oxidative stress, exhibiting significant reductions in cell viability in comparison to controls at 250 µM, 500 µM and 1 mM H_2_O_2_. The G37R mutation showed the greatest resistance to the stress, with significantly increased viability in comparison to the G93A and H48Q mutations. Data presented as mean with SD (n = 5). Statistical analyses by two-way ANOVA with Bonferroni post-test, all significant values presented are P≤0.01. (Key A-C graphs: ▪ = pIRES v G93A, α = pIRES v H48Q, ▪ = WTSOD1 v pIRES, # = WTSOD1 v G93A, λ = WTSOD1 v H48Q, ✓ = WTSOD1 v H48Q, ★ = H48Q v G93A, ∞ = G37R v pIRES, + = G37R v WTSOD1, ▪ = G37R v G93A, X = G37R v H48Q). D-F. LDH cytotoxcity assays. LDH release was measured over two-10 hours at D.250 µM. E 500 µM. F.1 mM H_2_O_2._ The G93A mutation generally showed the greatest LDH release compared to the controls and G37R/H48Q mutations. The G37R mutation showed a similar LDH release to that of controls across all time points and stress conditions. Data presented as mean with SD (n = 4). Statistical analyses by two-way ANOVA with Bonferroni post-test, **** = P<0.0001, *** = P<0.001, ** = P<0.01, * = P<0.05.

The effect of oxidative stress on cell viability was investigated in controls and NSC34s expressing G93A, G37R, or H48Q human mutant SOD1 as described previously. Little difference in cell viability across all cell lines was seen with 50 µM and 100 µM H_2_O_2_ treatment (data not shown). Differential vulnerability to oxidative stress in the G93A mutation was evident when cells were treated with 250 µM H_2_O_2,_ which was observed both in comparison to the controls and the other mutations (Figure 6A–C). Exposure to 500 µM and 1 mM H_2_O_2_ produced a reduction in viability such that SOD1 mutant lines displayed rapid induction of cell death. The G93A mutation displayed a significant reduction in cell viability with a 500 µM H_2_O_2_ exposure in comparison to both controls and the other mutations (p≤0.05), and the H48Q mutation showed a significant reduction in viability in comparison to controls at six and ten hours (p≤0.01) (Figure 6B). The G37R mutation showed a steady decline over time with both 500 µM and 1 mM treatments, but retained the greatest survival at ten hours (Figure 6C) and showed significantly greater viability than the other mutations (p≤0.001). Prolonged treatment with H_2_O_2_ (>six hours) would be expected to significantly reduce viability across all cell types. The G93A mutant NSC34 cells were the most severely affected, together with the H48Q mutant cells, as time and H_2_O_2_ concentration increased. In contrast, the G37R mutant cells displayed greater viability across all concentrations at all the investigated time points (p≤0.05). The pIRES vector and WTSOD1 controls showed reduction in viability but these did not become pronounced until persistent exposure to high concentrations of H_2_O_2_.

To further investigate the effect of oxidative stress on cellular injury, lactate dehydrogenase (LDH) release from the cell was measured to quantitatively measure cell lysis in the presence of H_2_O_2._ No difference in LDH release was observed between mutants and controls following a two-hour treatment with 250, 500 and 1 mM H_2_O_2_ (Figure 6 D–F). Treating with 250 µM H_2_O_2_ for four and six hours, led to a significant increase in LDH release in the G93A mutant compared to the control and H48Q/G37R mutant cell lines (p≤0.01). A similar trend was also observed when treating with 500 µM and 1 mM H_2_O_2._ The G37R mutant cells displayed comparable LDH release to the controls at the time points and H_2_O_2_ tested, which was consistent with the trypan blue cell viability data following H_2_O_2_ treatment (Figure 6 A–C). The H48Q mutant cells in general showed greater LDH release than controls at 250 µM H_2_O_2_. However at higher concentrations no differences were observed between the mutant and controls.

### Comparing mitochondrial bioenergetics of NSC34 cells expressing different mutant SOD1 transgenes

**Figure 7:**
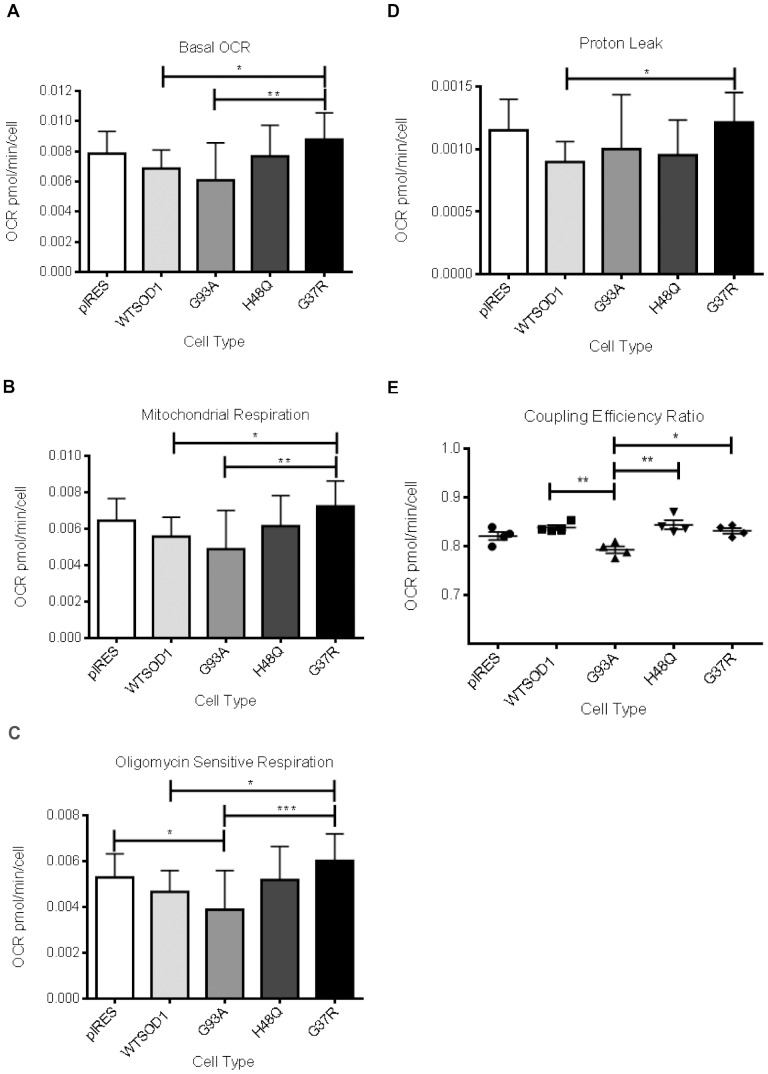
The effect of the SOD1 mutation on cellular respiration. A. Basal OCR (bOCR). B. Mitochondrial OCR (mOCR) calculated by subtracting OCR in the presence of rotenone from bOCR. The G37R mutation showed significantly higher bOCR and mOCR than WTSOD1 and G93A SOD1. C. Oligomycin sensitive respiration (coupled mitochondrial respiration). The rate of ATP turnover (coupled respiration) in a basal state can be determined from the decrease in OCR in the presence of oligomycin, which is displayed here as oligomycin sensitive respiration. The G93A mutation showed significantly reduced coupled respiration in comparison to the pIRES vector and G37R mutant cells. D. Mitochondrial proton leak. OCR in the presence of rotenone subtracted from OCR in the presence of oligomycin determines proton leak. G37R showed significantly increased proton leak compared to G93A and WTSOD1. E. Coupling efficiency (CE) ratio. Calculated by dividing the coupled respiration by mOCR. G93A SOD1 showed significantly reduced CE ratio compared to the SOD1 mutations and WTSOD1. Data presented as mean with SD (n = 4). Statistical analyses by one-way ANOVA with Bonferroni post-test, *** = P<0.001, ** = P<0.01, * = P<0.05.

Metabolic assays as described for the G93A mutation were carried out in the additional SOD1 mutant cells to compare mitochondrial bioenergetics between different SOD1 mutations. The experimental set-up was identical to that for the G93A mutant SOD1 versus control assays, with the controls and G93A mutant SOD1 cells included. The results for the G93A mutation replicated our original findings, a non-significant (p>0.05) reduction in bOCR was observed between the G93A mutant and control cells. However, differences were observed between the mutations. bOCR was significantly lower in the G93A mutant cells in comparison to the G37R mutant cells (p≤0.01) (Figure 7A), indicating different effects of SOD1 mutations on mitochondrial metabolism. G37R mutant cells also showed higher bOCR than WTSOD1, this was shown to be mitochondrial specific as when rotenone was used to determine the fraction of cellular oxygen consumption linked to the mitochondria, the G37R mutant cells showed significantly higher (p≤0.01) mitochondrial respiration compared with WTSOD1 and G93A SOD1 cells (Figure 7B). The spare respiratory capacity of the G93A cells was reduced compared to the other SOD1 mutations and controls (as shown previously), however the reduction was not significant (p>0.05, data not shown).

The rate of mitochondrial ATP synthesis (ATP turnover) was investigated by the application of oligomycin. The addition of oligomycin shifts the entire cellular ATP synthesis towards glycolysis so that subtraction of the post-treatment OCR from bOCR indicates oligomycin sensitive respiration (coupled respiration, Figure 3A). Overexpression of the different SOD1 mutations produced different results in terms of mitochondrial-coupled respiration. The G93A mutant cells showed significantly lower ATP turnover (p≤0.01) in comparison to the pIRES vector but not WTSOD1 (Figure 7C), whilst the H48Q mutation showed no difference in coupled respiration compared to controls. The G37R mutation, however, showed significantly increased coupled respiration compared to WT (p≤0.05) and G93A SOD1 (p≤0.001).

During ATP production, a percentage of protons leak across the inner mitochondrial membrane. These protons are able to pass back into the mitochondria, and in the absence of ATP synthesis the proton circuit is largely completed by proton leak. Proton leak can be determined by subtracting the respiration rate after the application of rotenone from the oligomycin sensitive respiration rate. No difference was observed between the G93A and H48Q mutations and controls in terms of proton leak (Figure 7D). However, the G37R mutant cells showed a significantly greater (p≤0.05) proton leak in comparison to the WTSOD1 cells. Dysfunctional mitochondria are expected to show an increase in proton leak as much of their energy generation is linked to uncoupled respiration. Coupling efficiency ratio (CE ratio) is a useful indicator of mitochondria dysfunction, as it is sensitive to changes in mitochondrial bioenergetics and is an internally normalised ratio [28]. Calculated by dividing the coupled respiration by mOCR, CE ratio was significantly reduced in G93A cells compared to WTSOD1 and the H48Q and G37R SOD1 mutations (Figure 7E).

Difference in glycolytic flux between the mutations was also investigated; however, no significant differences were observed for basal ECAR between the controls and the mutations (data not shown). The induction of ECAR in the presence of the mitochondrial inhibitors oligomycin, FCCP and rotenone was also measured to assess the glycolytic capacity of the cells. However, there was no significant difference in glycolytic capacity (p>0.05) between controls and mutant cells lines (data not shown).

### Effect of Oxidative stress on Metabolic Function in NSC34 Cells

**Figure 8:**
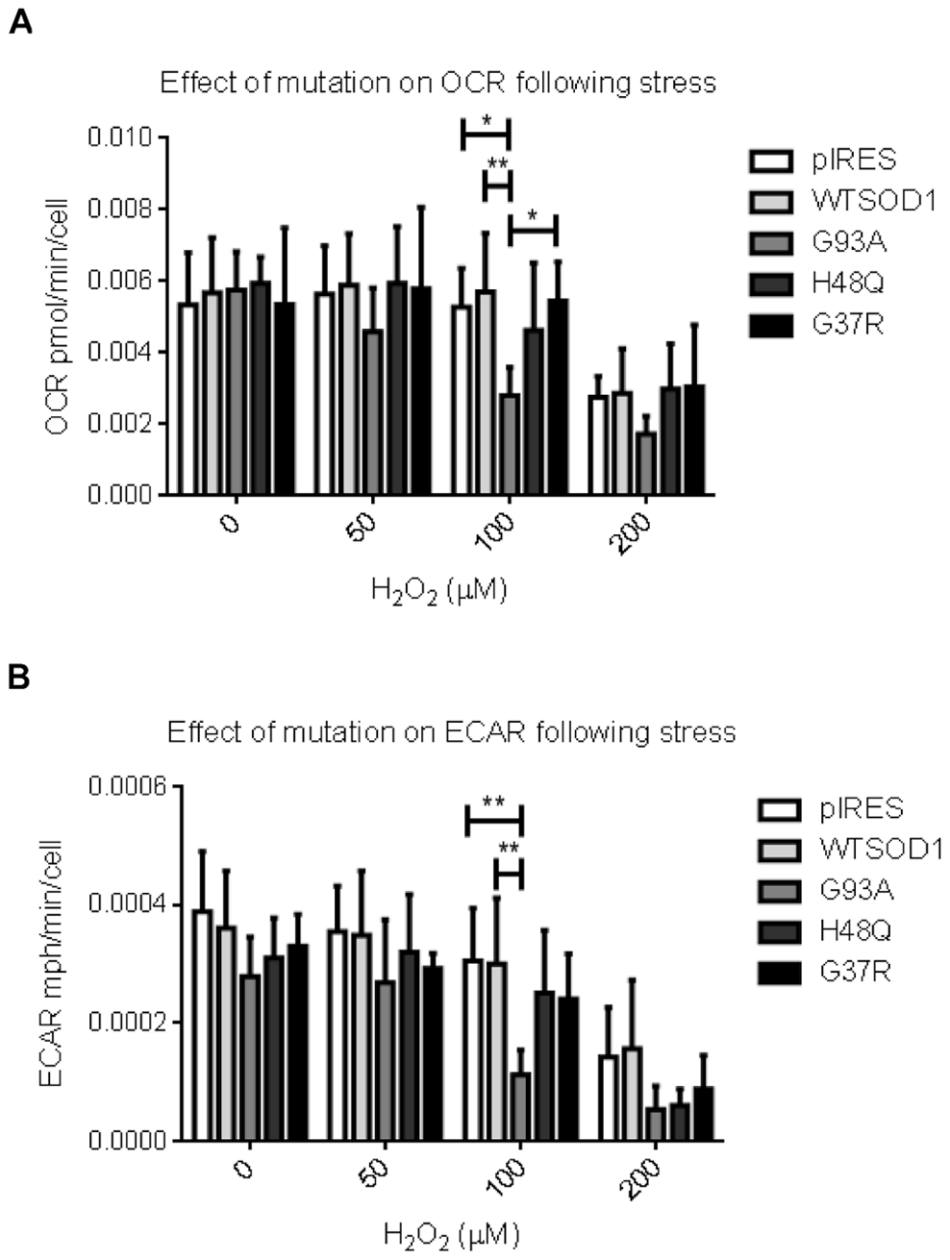
The effect of oxidative stress on mitochondrial and metabolic function. A. bOCR was measured following H_2_O_2_ stress. The G93A mutation had significantly reduced oxygen consumption in comparison to WTSOD1 and the G37R mutant human SOD1 after 100 µM stress. At 200 µM all cells displayed diminished basal OCR. B. ECAR was measured following H_2_O_2_ stress. After H_2_O_2_ treatment the G93A mutation displayed a significant reduction in ECAR at 100 µM in comparison to both the vector and human SOD1 controls. Data presented as mean with SD (n = 6), statistical analyses by two-way ANOVA with Bonferroni post-test, ** = P<0.01, * = P<0.05.

To assess the effect of SOD1 mutation on the metabolic susceptibility to oxidative stress, the cell lines were subjected to three sub-lethal doses of H_2_O_2_ (up to 200 µM for one hour) to induce oxidative stress and to determine whether these conditions introduced significant metabolic defects. Increasing levels of H_2_O_2_ led to a reduction of OCR in all cell lines (Figure 8A). However, a significant reduction in basal OCR was observed for the G93A mutant cells in comparison to the pIRES (p≤0.05), WTSOD1 (p≤0.01) and the G37R mutant cells (p≤0.05) following treatment with 100 µM H_2_O_2_ (Figure 8A).

Basal ECAR was unaffected in the control cells at 50 µM and 100 µM. However the G93A mutant cells showed a significant reduction in ECAR at 100 µM (p≤0.01) in comparison to both the pIRES and WTSOD1 controls (Figure 8B), which may indicate a susceptibility to oxidative stress caused by this particular mutation. A reduction in ECAR was observed for both controls and mutants at 200 µM. This data indicate that the G93A SOD1 mutation, unlike the G37R and H48Q mutations, confers a metabolic susceptibility to oxidative stress, not only in terms of cellular respiration but also glycolytic flux. 3-D Z-stack confocal analysis of mitochondria morphology using rhodamine 123 (as detailed in [29]) showed no significant differences between the G93A SOD1 mutation and controls under both basal conditions and following exposure to 100 µM H_2_O_2_ stress (data not shown).

## Discussion

Defective respiratory chain function associated with oxidative stress has previously been investigated in tissue from ALS patients and in mutant SOD1 models, supporting its involvement in the pathogenesis of this disease [30], [31], [32], [33], [34]. Here, using a motor neuron-like cell model transfected with various human mutant *SOD1* transgenes to investigate the impact of oxidative stress on cell viability and metabolic function, we show that the presence of SOD1 mutations affects cell viability in response to oxidative stress and impacts on metabolic function, with differences observed between different SOD1 mutations. Cellular dysfunction through oxidative stress plays a key role in MND disease progression. RT-qPCR demonstrated equivalent levels of transfection for the human wild-type and mutant *SOD1* transgenes in NSC34s, at the level of transcription. This data demonstrates the differences in susceptibility to oxidative stress and mitochondrial function identified in this study are not simply due to an over-expression of the mutant transgene.

The G93A and G37R mutations are confined to the β-strands of Cu/Zn SOD1, whereas the H48Q mutation is located in one of the histidine residues that coordinate copper, reducing its affinity for the ion. H48Q is superoxide dismutase inactive in comparison to the G93A and G37R mutations, which display typical enzymatic activity and bind copper in a coordination environment similar to that of WTSOD1 [25], [35], [36], [37]. Exposure to oxidative stress revealed significant differences in terms of susceptibility to cell death between the controls and SOD1 mutations, with the G93A mutant cells showing the greatest susceptibility (Figure 1 and Figure 6). H48Q mutant cells were also increasingly vulnerable following periods of more prolonged exposure to stress. The G37R mutant cells, in contrast, showed an exponential reduction in cell viability over time with increasing doses of hydrogen peroxide, but maintained cell viability at a level equal to or in some cases above that of the controls as shown by trypan blue exclusion and the LDH assay (Figure 6).

There is evidence from both cellular and animal models of ALS suggesting that SOD1 mutation leads to varying levels of cellular toxicity depending on the mutation in question. ALS-associated SOD1 mutations show the propensity to aggregate both with there-self and other proteins, which may be a result of disruption to the native protein folding [38]. A study investigating the correlation between the propensity for aggregation and conformational stability of SOD1 showed G93A to have the highest conformation instability, and therefore more prone to aggregation, in comparison to other SOD1 mutations, including G37R [39]. Another study also highlighted the differences between the mutations in transgenic mouse models of ALS. The G93A mice exhibited the fastest disease onset and had the shortest lifespan in comparison to the G37R and H46R/H48Q mutations [40]. The G37R mutation also seemed be less prone to forming insoluble aggregates than the G93A mutation. This may be part of the reason why the G93A mutation appeared more toxic in our study.

Dynein is crucial for axonal transport of mitochondria; aggregated G93A SOD1 has been shown to bind to dynein and this interaction has been proposed to facilitate the formation of aggresome-like inclusions in the cell [41]. Interestingly, an H48Q SOD1 mutation displayed a lower affinity for dynein compared to G93A and was less prone to forming high molecular weight complexes and large inclusions than the more toxic G93A mutation.

Here we show the G93A mutation to be more susceptible to oxidative stress induced cell death and mitochondrial dysfunction, which may be a result of the increased toxicity of this mutation as outlined here. In addition to this, G93A SOD1 have been shown to bind mitochondria within the spinal cord of transgenic mouse models of disease, forming high molecular weight aggregates, which are subsequently bound by apoptotic regulator Bcl-2 [30]. The sequestration of Bcl-2 by mutant SOD1 aggregates will render the protein non-functional, and inhibition of Bcl-2 binding to pro-apoptotic proteins may reduce cellular viability. Bcl-2 is also important for maintaining the mitochondrial membrane potential [42], therefore its sequestration in to SOD1 aggregates may lead to disruption of the mitochondrial membrane potential as observed in this study (Figure 5). G37R SOD1 were also shown to bind Bcl-2, but to a lesser degree [30].

Variations between the individual mutations likely underlie the differences seen in susceptibility to oxidative stress in these experiments. Phenotypic heterogeneity is also seen between patients with different *SOD1* mutations [43], adding further complexity to studying the pathology of the disease. The rapid induction of cell death following exposure to 500 µM and 1 mM concentrations of H_2_O_2_ may indicate a sudden loss of, as-yet undefined, compensatory mechanisms and an inability of the cells to recover from the insult. The cells may be able to compensate at lower concentrations, and hence only show minor reduction in viability. The redox system is part of a complex signaling network. Previous studies have shown that low concentration of ROS can influence the regulation of intracellular signaling such as the phosphoinositide 3-kinase (PI3K) pathway and the mitogen activated protein kinase (MAPK) cascade [44], [45] and which potentially explain the maintained viability observed with moderate H_2_O_2_ treatment. Previously published gene expression data from our laboratory showed a greater downregulation of key pentose phosphate metabolic enzyme activity in the G93A mutant SOD1 NSC34 cells compared to WTSOD1 and G37R [22]. The downregulation of the antioxidant response in G93A mutant cells coupled with a high propensity of the mutant to aggregate in the mitochondria may lead to higher ROS levels and increased susceptibility to mitochondrial dysfunction and cell death in the G93A SOD1 cells.

We also investigated the bioenergetic capacity of the NSC34 cells, to determine if the presence of disease-causing mutations had differential effects on mitochondrial function that could be linked to susceptibility to cell death under oxidative conditions. The XF24 Seahorse Bioanalyser simultaneously measures aerobic respiration and glycolysis within intact cells, and has previously been used to investigate mitochondrial bioenergetics in Alzheimer’s and Parkinson’s diseases [46], [47], [48]. Under basal culture conditions, no extensive metabolic defects were observed between controls and SOD1 mutants; the differences were subtle, with the G93A mutation showing the greatest dysfunction. Although no significant bOCR or mOCR defects were observed, the G93A mutation showed reduced mitochondrial coupled respiration compared to the pIRES and G37R cell lines (but not WTSOD1, Figure 7 A–C). In addition, reduced coupling efficiency ratio was observed for the G93A mutation compared to WTSOD1 and the H48Q and G37R SOD1 mutations (Figure 7E). This indicates differences in the efficiency of oxidative phosphorylation between mutations and could explain why the G93A mutant cells show a reduced mitochondrial membrane potential (Figure 5) and increased susceptibility to oxidative stress in comparison to the G37R mutant (Figure 6). Interestingly, the G37R SOD1 mutation showed increased mitochondrial coupled respiration compared to G93A and WTSOD1 but also increased proton leak compared to the latter (Figure 7D). Small changes in uncoupling can have physiological benefits by decreasing the mitochondrial membrane potential and subsequently reducing ROS production, however, large changes usually indicate damaged mitochondria [28]. It must be noted that treatment with oligomycin leads to slight mitochondrial hyperpolarization, which may over-estimate the proton leak and under-estimate ATP turnover; however in most cell types the error is relatively small [49].

Previous work using the NSC34 cell model identified significant reduction in the activity of complex II and IV of the mitochondrial respiratory chain in cells transfected with G93A or G37R mutant SOD1, in comparison to control vector-only cells [14]. No significant differences were seen for complex I and III activity. Defects in the mitochondrial membrane potential in G93A SOD1 transfected SH-SY5Y cells have also been observed [50]. However, another study found the activities of mitochondrial ATP synthesis, cytochrome c oxidase, and citrate synthase were unchanged in cells expressing G93A or G85R mutant SOD1 in comparison to control cells [51], indicating not only the variability in using different cell models to study disease but also the complex multi-factorial nature of the disease and how multiple factors are likely to influence functional capacity. The lack of significant reduction in total mitochondrial respiration observed in this study may be due to the fact that we assessed mitochondrial function in real time using intact cells, which gives greater physiological relevance than measuring isolated mitochondrial complex activity.

The effect of oxidative stress on the bioenergetic profile of the cells was investigated and significant differences in measurements of ECAR and OCR were observed between the mutant-transfected cell lines, again reflecting differences between mutations (Figure 8). These assays were performed under sub-lethal stress conditions (50 µM to 200 µM H_2_O_2_ for one hour). Treatment with similar or lower doses for longer periods (although not severe enough to induce cell death) may show different responses of the mutants over time. Overall, the data suggest that overexpression of the SOD1 G93A mutation renders the neuronal cells not only more susceptible than controls to oxidative stress in terms of cell survival, but in terms of increased susceptibility to perturbations of mitochondrial respiration and glycolytic metabolism, since this was the only mutation to show significant reductions in ECAR and OCR after H_2_O_2_ treatment.

The G93A SOD1 neuronal model has previously been shown to have higher basal cellular oxidative stress than WTSOD1 and to be more susceptible to serum withdrawn oxidative stress in terms of cell survival [14], [52]. Downregulation of key metabolic enzymes in the pentose phosphate pathway, which produce NADPH, essential for the cells antioxidant capacity, coupled with dysregulation of the antioxidant transcription factor NRF-2, may explain why G93A cells are more susceptible to H_2_O_2_ induced oxidative stress than vector alone or WTSOD1 overexpression [22]. In addition to the defect in bOCR, we show here that the G93A SOD1 cells have an increased metabolic susceptibility to oxidative stress in terms of glycolytic flux. This, along with a previous study showing that inhibition of glycolysis has a detrimental effect on cell viability in G93A NSC34 cells [14] suggests that oxidative stress not only leads to mitochondrial dysfunction in ALS but also dysregulates key metabolic pathways such as glycolysis. Increases in glycolytic flux would be crucial to compensate for the energy deficit produced by mitochondrial dysfunction and protect the neurone from oxidative stress induced cell death. Other *in vitro* investigations suggest depletion of intracellular NAD pools and/or of the inactivation of the glycolytic enzyme glyceraldehyde-3-phosphate dehydrogenase (GAPDH), due to oxidative stress, leads to disruption of glycolysis [53], [54]. Whilst the H_2_O_2_ generated by the neurotoxin 6-hydroxydopamine has been shown to lead to the loss of glycolytic activity via lipid peroxidation and inhibition of lactate dehydrogenase in neuroblastoma N2-A cells [55].

Here we have applied a novel technique to investigate metabolic function within intact motor neuronal NSC34 cells under basal and oxidative stress conditions. Differences in cellular metabolic and bioenergetic function between the mutations are consistent with the differences observed in viability. The G93A mutation was particularly susceptible to oxidative stress in terms of cell survival and mitochondrial dysfunction, demonstrating significantly lower OCR, spare respiratory capacity and mitochondrial respiration in comparison to the G37R mutation, which was the least susceptible to oxidative stress under the conditions investigated. Additionally, G93A was the only SOD1 mutation to show significant changes in OCR and ECAR under stress conditions. The H48Q mutation lay between the G93A and G37R mutations in relation to mitochondrial bioenergetic capacity and susceptibility to oxidative stress. This work contributes to the growing field of mitochondrial bioenergetic dysfunction in motor neuron disease and future work will address the underlying mechanisms by which these changes occur.

## Supporting Information

Figure S1 **Transfection level of the human mutant SOD1 transgenes were investigated by RT-qPCR.** Comparison of the difference in threshold cycle (ΔCT) between the human *SOD1* transgene (SOD1) and a reference mouse *Sod1* gene (Sod1) showed no significant differences in the level of human SOD1 between the cell lines. Data presented as mean with SD (n = 3), statistical analyses by one-way ANOVA with Bonferroni post-test.
(TIF)
