# Supplementary figures and images for: The Effect of SOD1 Mutation on Cellular Bioenergetic Profile and Viability in Response to Oxidative Stress and Influence of Mutation-Type
Source: PLoS One. 2013 Jun 28;8(6):e68256. doi: 10.1371/journal.pone.0068256 (PMC3695905; doi:10.1371/journal.pone.0068256)

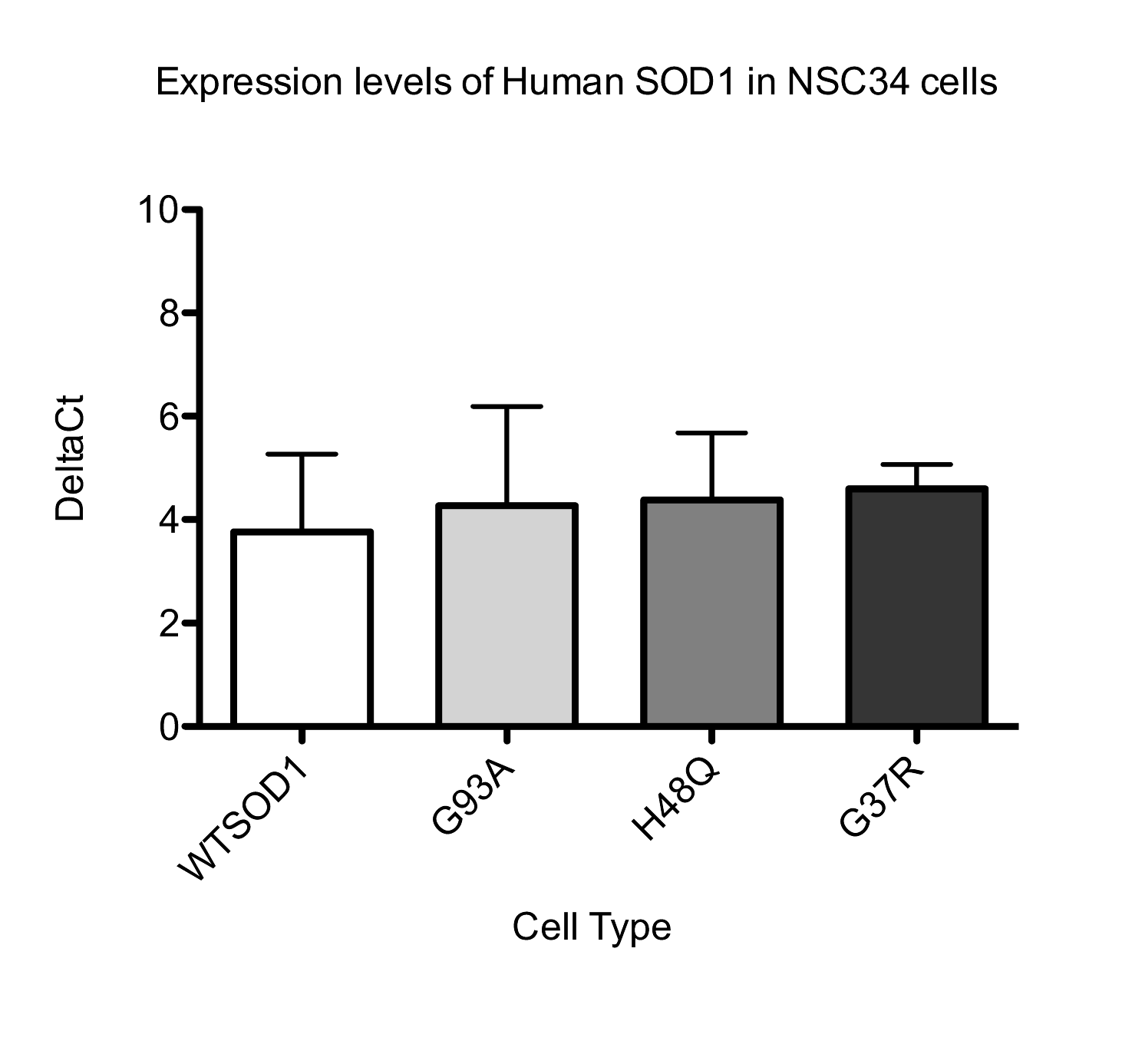

Supplement: Figure S1 — Transfection level of the human mutant SOD1 transgenes were investigated by RT-qPCR. Comparison of the difference in threshold cycle (ΔCT) between the human SOD1 transgene (SOD1) and a reference mouse Sod1 gene (Sod1) showed no significant differences in the level of human SOD1 between the cell lines. Data presented as mean with SD (n = 3), statistical analyses by one-way ANOVA with Bonferroni post-test. (TIF) [file pone.0068256.s001.tif]
